# Stability Analysis of Mathematical Model including Pathogen-Specific Immune System Response with Fractional-Order Differential Equations

**DOI:** 10.1155/2018/7930603

**Published:** 2018-12-04

**Authors:** Bahatdin Daşbaşı

**Affiliations:** Kayseri University, Faculty of Applied Sciences, TR-38039 Kayseri, Turkey

## Abstract

In this study, the mathematical model examined the dynamics between pathogen and specific immune system cells (memory T cells) for diseases such as chronic infection and cancer in which nonspecific immune system cells are inadequate to destroy the pathogen and has been suggested by using a system of the fractional-order differential equation with multi-orders. Qualitative analysis of the proposed model reveals the equilibrium points giving important ideas about the proliferation of the pathogen and memory T cells. According to the results of this analysis, the possible scenarios are as follows: the absence of both pathogen and memory T cells, only the existence of pathogen, and the existence of both pathogen and memory T cells. The qualitative analysis of the proposed model has expressed the persistent situations of the disease where the memory T cells either do not be able to respond to the pathogen or continue to exist with the disease-causing pathogen in the host. Results of this analysis are supported by numerical simulations. In the simulations, the time-dependent size of the tumor population under the pressure of the memory T cells was tried to be estimated.

## 1. Introduction

For three centuries, the theory of fractional derivatives was developed as a pure theoretical field of mathematics, useful only for mathematicians. But, the use of fractional-orders differential and integral operators in mathematical models has become increasingly common of late years. Therefore, various forms of fractional-order differential equations are suggested for standard models. In this sense, the fractional-order calculus plays an important role in physics [1], thermodynamics [2], viscoelasticity [3], electrical circuits theory [4], fractances [5], mechatronics systems [6], signal processing [7], chemical mixing [8], chaos theory [9], engineering [10], biological system [11], and other applications [12]. Also, a large number of literatures on the application of fractional-order differential equations (FODEs) in nonlinear dynamics have been improved. Especially, when the biological applications of FODEs have considered, it is a rich source for mathematical ideas [13].

The mathematical modeling of diseases in biological applications is a subject discussed in the literature. Such models are considered under two main headings as by modeling the size of the spread of infected individuals in a population (SIR) and modeling the population size of the pathogens such as the tumor in an individual as it is here. The word tumor simply refers to a mass. This is a general term that can refer to benign (generally harmless) or malignant (cancerous) growths. Many types of tumors are considered to be a major factor in many fatal diseases in human history. Fundamentally, it is said that this disease is a complex process for both tumor and host. Although different treatment strategies are proposed for tumors, the first and foremost role in disease progression belongs to the immune system of the individual (or host) [14]. The immune system is stated as a system of biological structures and processes in an organism that protects the body from the possible hazardous organism by recognizing and responding to antigens. In more detail, the immune system cells such as T cells are generally described in terms of two different types. These are the effector and the memory of T cells. The ordinary behaviour of the immune system is generally an acute infection, controlled initially by effector T cells (aspecific response or the innate immune system response), later by memory T cells (specific response or the adaptive immune response), until complete clearance of the pathogen. The adaptive immune response is much slower to respond to threats and infections than the innate immune response, which is primed and ready to fight at all times [15]. Especially, T cells are a crucial component of the adaptive immune response against malignancies. Antigen-experienced T cells specific for tumor antigens can be recovered from the blood, lymphoid organs, and tumors of both cancer patients and tumor-bearing mice [16]. Concordantly, the reactions of different hosts in case of the same disease may be different because of the immune system response given by host, and so, the disease progression varies from person to person. Within this context, dynamics of relevances between immune systems cells (T cells) and tumor are significant to find out the nature of the disease. The problem is to try to obtain the known biological features without making the mathematics too complicated.

The basic of a most useful explanation of fractional calculus is memory concept. If the output of a system at each time *t* depends only on the input at time *t*, then such systems are said to be memoryless systems. On the contrary, if the system has to remember previous values of the input in order to determine the current value of the output, then such systems are said memory systems [17, 18]. Accordingly, the behaviour of most biological systems has memory or after-effects. The modeling of these systems by FODEs has more advantages than classical integer-order modeling, in which such effects are neglected. Also, FODEs are, at least, as stable as their integer order counterpart [11]. In the process of modeling real-life situations, the created mathematical models by using the fractional-order differential operations allow to display the some extra cases regarding the stability region of the equilibrium point of the mathematical model caused by parameters such as derivative orders. For this reason, the mathematical models formed by FODEs are more realistic and feasible [19]. Additionally, stability analysis of equilibrium points for mathematical models consisting of FODEs with multi‐orders and its systems is more general than those of the same-orders too.

Although there are many studies that examined the dynamics between tumor and immune system response, the proposed model in this study differs from them in terms of both mathematical structure such as the use of Holling function type-2 (functional and numerical responses) in the model consisting of the FODE system with multi-orders and examination of qualitative analysis of the proposed model. In this sense, it was tried to bring a different perspective from the previous studies.

In this study, a FODE model with multi-orders considering the basic mechanisms of tumor and the memory T cells having functional and numerical responses, respectively, has been constructed, and so, the qualitative analysis of the proposed model was performed. The reason for using the Holling function type-2 is to show the limit cycle behaviour of system [20]. The certain conditions dependent on the development of the tumor population under the pressure of memory T cells was obtained. In this respect, all of the possible scenarios related to the tumor size were tried to be explained as parameter-dependent. Additionally, numerical analysis of the model was given as to be compatible with the qualitative analysis.

## 2. Preliminaries and Definitions

In here, the main definitions and properties of fractional derivative operators have been expressed. Also, the FODE systems with multi-orders have been introduced, and the properties such as stability and existence of the equilibrium points of such systems are given.

### 2.1. Fractional Differential Operators

There are various definitions of a fractional derivative with the order *α* > 0. The definitions of Riemann–Liouville and Caputo are used most widely. The Caputo sense was used in this study. Taking into account the definition of Caputo sense, the fractional derivative of the function *f*(*t*) is identified as

(1) $$\begin{array}{c} D^{\alpha }f\left(t\right)=J^{m-\alpha }D^{m}f\left(t\right)=\frac{1}{\Gamma \left(m-\alpha \right)}\int_{0}^{t}\frac{f^{\left(m\right)}\left(\tau \right)}{{\left(t-\tau \right)}^{\alpha -m+1}}d\tau , \end{array}$$

for *m* − 1 < *α* ≤ *m*, *m* ∈ *ℕ*, *t* > 0 [21].

### 2.2. The FODE System with Multi-orders

Let us consider that *t* is the time parameter. We have assumed that the system of FODE with multi-orders is given as the following equation:

(2) $$\begin{array}{c} D_{t}^{\alpha }X\left(t\right)=F\left(t,X\right),X\left(0\right)=X_{0}, \end{array}$$

where the variable *X*=[*x*_1_1__(*t*), *x*_2_1__(*t*),…,*x*_*n*_1__(*t*)]^*T*^ ∈ *ℝ*^*n*^, the initial conditions by *X*_0_=[*x*_1_0__(0), *x*_2_0__(0),…,*x*_*n*_0__(0)]^*T*^ ∈ *ℝ*^*n*^, the functions by *F*=[*f*_1_, *f*_2_,…,*f*_*n*_]^*T*^ ∈ *ℝ*^*n*^ and *f*_*i*_ : [0, +*∞*)*xℝ*^*n*^⟶*ℝ* for *i*=1,2,…, *n*, and the derivative orders by *α*=[*α*_1_, *α*_2_,…,*α*_*n*_]^*T*^.

Also, when it is considered as *D*_*t*_^*α*^=[*D*_*t*_^*α*_1_^, *D*_*t*_^*α*_2_^,…,*D*_*t*_^*α*_*n*_^]^*T*^, *D*_*t*_^*α*_*i*_^ indicates *α*_*i*_ th-order fractional derivative in the Caputo sense. In this sense, it is *D*_*t*_^*α*^*X*(*t*)=[*D*_*t*_^*α*_1_^*x*_1_1__(*t*), *D*_*t*_^*α*_2_^*x*_2_1__(*t*),…,*D*_*t*_^*α*_*n*_^*x*_*n*_1__(*t*)]^*T*^. The multi-orders can be mathematically any real or complex vector. In this study, the real case was only taken into account. Throughout the paper, we restrict *α*_*i*_ to a rational number in the interval (0,1] [22].

**Remark 1 .**

From (2), we have assumed that

(3) $$\begin{array}{c} F\left(t,X\right)=F\left(X\right), \end{array}$$

where the independent variable *t* is not clearly seen in the function *F*. The equilibrium point of (3) is the point $\bar{X}=\left(\bar{x_{1}},\bar{x_{2}},\ldots ,\bar{x_{n}}\right)$ obtained from the equations $F\left(\bar{X}\right)=0$.

**Remark 2 .**

For each equilibrium point $\bar{X}$ of the autonomous system in (3), the eigenvalues *λ* obtain from the following equation:

(4) $$\begin{array}{c} \det\left(\mathrm{diag}\left(\lambda^{m\alpha_{1}},\lambda^{m\alpha_{2}},\ldots ,\lambda^{m\alpha_{n}}\right)-J\left(\bar{X}\right)\right)=0, \end{array}$$

where $J\left(\bar{X}\right)$ is the Jacobian matrix evaluated at the equilibrium point and *m* is the smallest of the common multiples of the denominators of the rational numbers *α*_1_, *α*_2_,…, *α*_*n*_ [23].

**Theorem 1 .**

For each equilibrium point $\bar{X}$ of system (3), $\bar{X}$ is locally asymptotically stable (LAS), if the eigenvalues *λ*_*i*_ obtained from (4) satisfy Routh–Hurwitz Stability Criteria or the inequalities |arg(*λ*_*i*_)| > (*π*/2*m*) for *i*=1,2,…, *m*(*α*_1_+*α*_2_). Here, *m* has been defined in Remark 2 [22]. Because the 2-dimensional of system (3) is used in the proposed model in this study, the stability analysis of such systems are described in detail below.

**Remark 3 .**

Let us assume that the autonomous system of FODE with multi-orders is as following:

(5) $$\begin{array}{c} D^{\alpha_{1}}x_{1}\left(t\right)=f_{1}\left(x_{1},x_{2}\right),\\ D^{\alpha_{2}}x_{2}\left(t\right)=f_{2}\left(x_{1},x_{2}\right), \end{array}$$

with the nonnegative initial conditions

(6) $$\begin{array}{c} x_{1}\left(0\right)=x_{\text{o}1}\;\;\mathrm{and}\;\;x_{2}\left(0\right)=x_{\text{o}2}, \end{array}$$

where the derivative orders *α*_1_ and *α*_2_ are rational numbers in the interval (0,1]. The equilibrium point of system (5) is the point $\bar{X}=\left(\bar{x_{1}},\bar{x_{2}}\right)$ obtained from the equations *D*^*α*_*i*_^*x*_*i*_(*t*)=0 for =1,2. To evaluate locally asymptotically stability (LAS) of equilibrium point, the Jacobian matrix, $J=\left[\begin{array}{cc} \partial f_{1}/\partial x_{1} & \partial f_{1}/\partial x_{2}\\ \partial f_{2}/\partial x_{1} & \partial f_{2}/\partial x_{2} \end{array}\right]=\left[\begin{array}{cc} {\left(f_{1}\right)}_{x_{1}} & {\left(f_{1}\right)}_{x_{2}}\\ {\left(f_{2}\right)}_{x_{1}} & {\left(f_{2}\right)}_{x_{2}} \end{array}\right]$, is used. Considering Remark 2, the eigenvalues *λ*_*i*_ for *i*=1,2,…, *m*(*α*_1_+*α*_2_) are obtained from the following equation:

(7) $$\begin{array}{c} \det\left(\mathrm{diag}\left(\lambda^{m\alpha_{1}},\lambda^{m\alpha_{2}}\right)-J\left(\bar{X}\right)\right)=\left|\begin{array}{cc} \left(\lambda^{m\alpha_{1}}-\;{\left.{\left(f_{1}\right)}_{x_{1}}\right|}_{\left(\bar{x_{1}},\bar{x_{2}}\right)}\right) & -{\left.{\left(f_{1}\right)}_{x_{2}}\right|}_{\left(\bar{x_{1}},\bar{x_{2}}\right)}\\ -{\left.{\left(f_{2}\right)}_{x_{1}}\right|}_{\left(\bar{x_{1}},\bar{x_{2}}\right)} & \left(\lambda^{m\alpha_{2}}-\;{\left.{\left(f_{2}\right)}_{x_{2}}\right|}_{\left(\bar{x_{1}},\bar{x_{2}}\right)}\right) \end{array}\right|=0. \end{array}$$

Therefore, the characteristic equation for eigenvalues is

(8) $$\begin{array}{c} \lambda^{m\left(\alpha_{1}+\alpha_{2}\right)}-\lambda^{m\alpha_{1}}{\left.{\left(f_{2}\right)}_{x_{2}}\right|}_{\left(\bar{x_{1}},\bar{x_{2}}\right)}-\lambda^{m\alpha_{2}}{\left.{\left(f_{1}\right)}_{x_{1}}\right|}_{\left(\bar{x_{1}},\bar{x_{2}}\right)}+\left(\begin{array}{c} {\left.{\left(f_{1}\right)}_{x_{1}}\right|}_{\left(\bar{x_{1}},\bar{x_{2}}\right)}{\left.{\left(f_{2}\right)}_{x_{2}}\right|}_{\left(\bar{x_{1}},\bar{x_{2}}\right)}\\ -{\left.{\left(f_{1}\right)}_{x_{2}}\right|}_{\left(\bar{x_{1}},\bar{x_{2}}\right)}{\left.{\left(f_{2}\right)}_{x_{1}}\right|}_{\left(\bar{x_{1}},\bar{x_{2}}\right)} \end{array}\right)=0. \end{array}$$

If the eigenvalues *λ*_*i*_ for *i*=1,2,…, *m*(*α*_1_+*α*_2_) satisfy Routh–Hurwitz stability criteria or the conditions,

(9) $$\begin{array}{c} \left|\arg\left(\lambda_{i}\right)\right|>\frac{\pi }{2m}, \end{array}$$

then the equilibrium point $\left(\bar{x_{1}},\bar{x_{2}}\right)$ is the LAS point for system (5).

**Figure 1:**
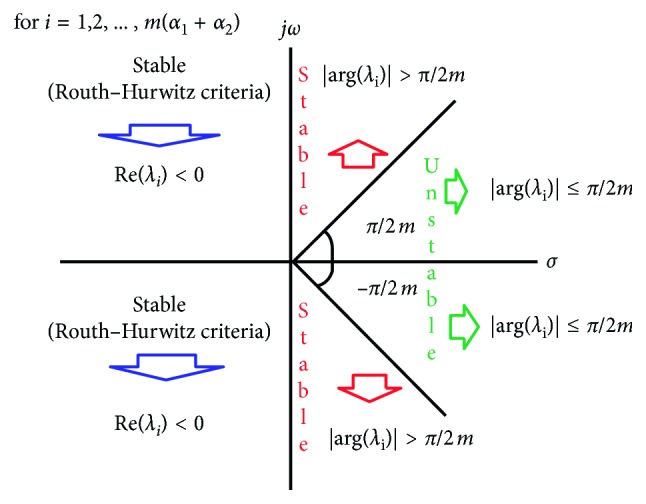
Stability region of the equilibrium point $\bar{X}$ of system (5).

For the system of FODE with multi-orders *α*_1_ and *α*_2_, the stability region is as shown in Figure 1 (where *σ* and *ω* are the real and imaginary parts of the eigenvalues, respectively, and $j=\sqrt{-1}$). By Figure 1, we openly see that the stability region of the equilibrium point of the FODE with multi-orders is greater than the stability regions of the integer-order case and the same fractional-order case [24].

**Remark 4 .**

Let *α*_1_=*α*_2_=*α* in system (5). In this case, we have the system

(10) $$\begin{array}{c} \begin{array}{c} D^{\alpha }x_{1}\left(t\right)=f_{1}\left(x_{1},x_{2}\right),\\ D^{\alpha }x_{2}\left(t\right)=f_{2}\left(x_{1},x_{2}\right), \end{array} \end{array}$$

with the nonnegative initial conditions *x*_1_(0)=*x*_o1_  and  *x*_2_(0)=*x*_o2_. From the equations *D*^*α*^*x*_*i*_(*t*)=0 for *i*=1,2, we have presumed that the equilibrium point of system (10) is $\bar{X}=\left(\bar{x_{1}},\bar{x_{2}}\right)$. If the eigenvalues *λ*_1_ and *λ*_2_ obtained from the equation

(11) $$\begin{array}{c} \text{Det}\left(J_{\left(x_{1},x_{2}\right)=\left(\bar{x_{1}},\bar{x_{2}}\right)}-\lambda I_{2}\right)=0 \end{array}$$

provide the conditions

(12) $$\begin{array}{c} \left(\left|\arg\left(\lambda_{1}\right)\right|>\frac{\alpha \pi }{2},\left|\arg\left(\lambda_{2}\right)\right|>\frac{\alpha \pi }{2}\right), \end{array}$$

then the equilibrium point $\left(\bar{x_{1}},\bar{x_{2}}\right)$ is the LAS point for system (10).

Conditions expressed in (12) can be detailed as the followings. Characteristic equation of (11) is the following generalized polynomial:

(13) $$\begin{array}{c} p\left(\lambda \right)=\lambda^{2}+a_{1}\lambda +a_{2}=0. \end{array}$$

When both the conditions (12) and the polynomial (13) are considered together, the conditions for LAS of the equilibrium point $\left(\bar{x_{1}},\bar{x_{2}}\right)$ are either Routh–Hurwitz conditions [25, 26]:

(14) $$\begin{array}{c} a_{1},a_{2}>0, \end{array}$$

or

(15) $$\begin{array}{c} a_{1}<0,4a_{2}>{\left(a_{1}\right)}^{2},\left|\tan^{-1}\left(\frac{\sqrt{4a_{2}-{\left(a_{1}\right)}^{2}}}{a_{1}}\right)\right|>\frac{\alpha \pi }{2}. \end{array}$$

## 3. Model Formulation

The proposed model is particularly well suited for describing diseases such as chronic infection and cancer in which the nonspecific immune system cells are inadequate to destroy the pathogen. Consequently, it has been proposed, and another extension of the models in [20, 27–33] has been analyzed.

It has been identified mathematically as a pathogen load, specifically tumor population, and level of memory T cells, namely, the adaptive immune response or specific response, in an individual. In this sense, we have assumed that the population densities of pathogen and memory T cells at time *t* are denoted by *P*(*t*) and *T*(*t*), respectively. Additionally, the memory T cells predate the tumor cells by a Holling function type-2.

Under the assumptions aforementioned, we have proposed the following system of FODE with multi-orders *α*_1_ and *α*_2_:

(16) $$\begin{array}{c} D_{t}^{\alpha_{1}}P=\beta_{P}P\left(1-\frac{P}{\Lambda }\right)-\frac{cP}{1+aP}T,\\ D_{t}^{\alpha_{2}}T=\frac{\mu P}{1+\nu P}T-\delta T,\\ 0<\alpha_{1},\alpha_{2}\leq 1, \end{array}$$

where *D*_*t*_^*α*_*i*_^ for *i*=1,2 indicates *α*_*i*_th-order fractional derivatives in the Caputo sense, it is *P*=*P*(*t*) and *T*=*T*(*t*), and the parameters have the following properties:

(17) $$\begin{array}{c} \beta_{P},\Lambda ,c,a,\mu ,\nu ,\delta \in R^{+}. \end{array}$$

In addition that, system (16) has to be finished with positive initial conditions *P*(*t*_0_)=*P*_0_ and *T*(*t*_0_)=*T*_0_. The parameters used in the model are defined as follows.

It is presumed that the pathogen follows a logistic growth rule with the carrying capacity Λ and the growth rate *β*_P_. The memory T cells proliferate proportionally to the pathogen load by the Holling function type-2. Since the pathogen capture rate of memory T cells is assumed to be proportional to the per capita growth rate of memory T cells, the constant *μ* represents the maximum growth rate for memory T cells and the constant *ν* is the pathogen population size at which the growth rate of memory T cells in half of its maximum. These situations are very suitable for the growth of memory T cells especially in case of chronic infection or tumor. Memory T cells have per capita natural death rate *δ*. Moreover, the pathogen die due to the action of the memory T cells, and we have presumed that the effect of these cells on pathogen is modeled using a saturating response, (*cP*/1+*aP*), subject to a maximum killing rate *c* and the level of memory T cells required for the half maximum effect, *a*.

**Remark 5 .**

Rate of replication of the immune system cells is higher than its death rate, at least every time the pathogen load is very high [20]. In this case, we have $\underset{t⟶\infty }{\lim}\left(\left(\mu P\left(t\right)\right)/\left(1+\nu P\left(t\right)\right)\right)=\mu /\nu$ by (16). Therefore, the following inequality is obtained:

(18) $$\begin{array}{c} \frac{\mu }{\nu }>\delta , \end{array}$$

by this limit.

**Proposition 1 .**

System (16) provides the followings. The free-disease equilibrium point *E*_0_(0,0) and the equilibrium point *E*_1_(Λ, 0), where only the pathogen exists and always exist. In addition to *E*_0_ and *E*_1_, there exists a third equilibrium point as *E*_2_=(*P*^*∗*^, *T*^*∗*^) for

(19) $$\begin{array}{c} P^{*}=\frac{\delta }{\mu -\nu \delta },\\ T^{*}=\frac{\beta_{P}\left(1-\left(P^{*}/\Lambda \right)\right)}{c/\left(1+aP^{*}\right)}, \end{array}$$

when (*δ*/(*μ* − *νδ*)) < Λ.

**Proof**

The steady states of the model (16) are again the intersection of null clines *D*^*α*_1_^*P*=0 and *D*^*α*_2_^  *T*=0 in (16). We have accepted that the solutions of theses equations consist of the pairs $\left(\bar{P},\bar{T}\right)$. Then, we have the following system:

(20) $$\begin{array}{c} \bar{P}\left(\beta_{P}\left(1-\frac{\bar{P}}{\Lambda }\right)-\frac{c}{1+a\bar{P}}\bar{T}\right)=0,\\ \bar{T}\left(\frac{\mu \bar{P}}{1+\nu \bar{P}}-\delta \right)=0. \end{array}$$

From the first equation of (20), it is $\bar{P}=0$ or $\beta_{P}\left(1-\left(\bar{P}/\Lambda \right)\right)-\left(c/1+a\bar{P}\right)\bar{T}=0$. Let $\bar{P}=0$, and then $\bar{T}=0$. Therefore, the system (16) has the free-disease equilibrium point *E*_0_(0,0). On the contrary, let $\beta_{P}\left(1-\left(\bar{P}/\Lambda \right)\right)-\left(c/1+a\bar{P}\right)\bar{T}=0$, that is, $\bar{T}=\left(\beta_{P}\left(1-\left(\bar{P}/\Lambda \right)\right)\right)/\left(\left(c/1+a\bar{P}\right)\right)$. If the value $\bar{T}$ is rewritten in the second equation of system (20), then we have found the equilibrium points *E*_1_(Λ, 0) and *E*_2_((*δ*/(*μ* − *νδ*)), (*β*_*P*_(1 − (*δ*/(*μ* − *νδ*))/Λ))/(*c*/1+*a*(*δ*/*μ* − *νδ*))). Considering (19), if *E*_2_ is rewritten, the point *E*_2_(*P*^*∗*^, *T*^*∗*^) is obtained. *E*_1_ always exists due to (17). Let us consider *E*_2_. *P*^*∗*^ is positive due to (17) and (18). On the contrary, *T*^*∗*^ is positive due to (17), when

(21) $$\begin{array}{c} \frac{\delta }{\mu -\nu \delta }<\Lambda . \end{array}$$

Therefore, we have a positive equilibrium point *E*_2_(*P*^*∗*^, *T*^*∗*^) where *P*^*∗*^ and *T*^*∗*^ are in (19).

In Table 1, biological existence conditions of equilibrium points of system (16) are showed.

**Proposition 2 .**

In system (16), let us consider derivative orders as

(22) $$\begin{array}{c} \alpha_{1}=\frac{k_{1}}{m_{1}},\alpha_{2}=\frac{k_{2}}{m_{2}}\text{and }k_{1},k_{2},m_{1},m_{2}\in Z^{+}, \end{array}$$

where the smallest common multiple of *m*_1_ and *m*_2_ is *m*. System (16) satisfies the following:

(a) *E*_0_(0,0) is a unstable point
(b) *E*_1_(Λ, 0) is LAS, when (*δ*/(*μ* − *νδ*)) > Λ. Also, if (*δ*/*μ* − *νδ*) ≤ Λ, then this point is a unstable point
(c) *E*_2_=(*P*^*∗*^, *T*^*∗*^) where *P*^*∗*^ and *T*^*∗*^ defined in (19) is LAS, when all roots *λ*_*i*_ for *i*=1,2,…, *m*(*α*_1_+*α*_2_) found from the equation
  (23) $$\begin{array}{c} \lambda^{m\left(\alpha_{1}+\alpha_{2}\right)}-\lambda^{m\alpha_{2}}\beta_{P}\left(A_{1}\left(A_{2}+1\right)-1\right)+\frac{\beta_{P}\delta^{2}A_{1}}{\mu \Lambda \left(1-A_{1}\right)}=0 \end{array}$$
  satisfy Routh–Hurwitz stability criteria or the condition |arg(*λ*_*i*_)| > (1/*m*)(*π*/2). In here, it is
  (24) $$\begin{array}{c} \left(1-\frac{P^{*}}{\Lambda }\right)=A_{1},\\ \left(\frac{aP^{*}}{aP^{*}+1}\right)=A_{2}. \end{array}$$

**Proof**

For the stability analysis of the equilibrium points, the functions in system (16) are assigned as

(25) $$\begin{array}{c} f\left(P,T\right)=P\left(\beta_{\text{P}}\left(1-\frac{P}{\Lambda }\right)-\frac{c}{1+aP}T\right),\\ g\left(P,T\right)=T\left(\frac{\mu P}{1+\nu P}-\delta \right). \end{array}$$

In this respect, the Jacobian matrix evaluated at each equilibrium point showed in Table 1 is

(26) $$\begin{array}{c} J\left(E_{i}\left(\bar{P},\bar{T}\right)\right)=\left(\begin{array}{cc} \beta_{P}\left(1-\frac{2\bar{P}}{\Lambda }\right)+\frac{\bar{T}c}{a\bar{P}+1}\left(\frac{a\bar{P}}{a\bar{P}+1}-1\right) & -\frac{c\bar{P}}{a\bar{P}+1}\\ \frac{\bar{T}\mu }{{\left(1+\nu \bar{P}\right)}^{2}} & \left(\frac{\mu \bar{P}}{1+\nu \bar{P}}-\delta \right) \end{array}\right), \end{array}$$

for *i*=0,1,2.

(a) For *E*_0_, the Jacobian matrix in (26) is
  (27) $$\begin{array}{c} J\left(E_{0}\left(0,0\right)\right)=\left(\begin{array}{cc} \beta_{P} & 0\\ 0 & -\delta \end{array}\right). \end{array}$$

Now, we will investigate whether the inequality (9) has been achieved. By (27), the eigenvalues have been found from the following determinant:

(28) $$\begin{array}{c} \det\left(\mathrm{diag}\left(\lambda^{m\alpha_{1}},\lambda^{m\alpha_{2}}\right)-J\left(E_{0}\left(0,0\right)\right)\right)=\det\left(\begin{array}{cc} \lambda^{m\alpha_{1}}-\beta_{P} & 0\\ 0 & \lambda^{m\alpha_{2}}+\delta \end{array}\right)=0. \end{array}$$

In this respect, the characteristic equation obtained from (28) is

(29) $$\begin{array}{c} \left(\lambda^{m\alpha_{1}}-\beta_{P}\right)\left(\lambda^{m\alpha_{2}}+\delta \right)=0. \end{array}$$

Thus, we have *λ*^*mα*_1_^=*β*_*P*_ and *λ*^*mα*_2_^=−*δ*. *λ*^*mα*_1_^ is real positive due to (17). Moreover, it is obtained as

(30) $$\begin{array}{c} \lambda_{i}=\sqrt[{\left(1/m\alpha_{1}\right)}]{\beta_{P}}\in R^{+}\;\text{for}\;\;i=1,2,\ldots ,m\alpha_{1}. \end{array}$$

For the eigenvalues in (30), it is arg(*λ*_*i*_)=0 for *i*=1,2,…, *mα*_1_. These eigenvalues are positive real number on the right side of the complex plane, and so, it is |arg(*λ*_*i*_)|=0 < (*π*/2*m*). Since the stability condition is not supplied, the equilibrium point *E*_0_(0,0) is a unstable point for system (16).

(b) From (24), the Jacobian matrix related to *E*_1_(Λ, 0) is
  (31) $$\begin{array}{c} J\left(E_{1}\left(\Lambda ,0\right)\right)=\left(\begin{array}{cc} -\beta_{P} & -\frac{c\Lambda }{a\Lambda +1}\\ 0 & \left(\frac{\mu \Lambda }{1+\nu \Lambda }-\delta \right) \end{array}\right). \end{array}$$

From the equation det(diag(*λ*^*mα*_1_^, *λ*^*mα*_2_^) − *J*(*E*_1_(Λ, 0)))=0, the characteristic equation of eigenvalues is

(32) $$\begin{array}{c} \left(\lambda^{m\alpha_{1}}+\beta_{\text{P}}\right)\left(\lambda^{m\alpha_{2}}-\left(\frac{\mu \Lambda }{1+\nu \Lambda }-\delta \right)\right)=0. \end{array}$$

Therefore, it is *λ*^*mα*_1_^=−*β*_*P*_ and *λ*^*mα*_2_^=((*μ*Λ/(1+*ν*Λ)) − *δ*). These equations are examined as the following:

1. *λ* ^*mα*_1_^ is a negative real number due to (17). By De Moivre's formula, we have *λ*^*mα*_1_^=*β*_*P*_*cisπ*⇒*λ*_*i*_=*β*_*P*_^(1/*mα*_1_)^*cis*(*π*/*mα*_1_)for *i*=1,2,…, *mα*_1_, such that $cis\pi =\cos\pi +i\sin\pi ,i=\sqrt{-1}$. Considering (9), the stability condition for *E*_1_(Λ, 0) is *α*_1_ < 2 due to |arg(*λ*)|=|*π*/(*mα*_1_)| > (*π*/2*m*). This condition has been always provided since 0 < *α*_1_, *α*_2_ ≤ 1 in (16).
2. On the other hand, we have considered the equation *λ*^*mα*_2_^=((*μ*Λ/(1+*ν*Λ)) − *δ*). If (*μ*Λ/(1+*ν*Λ)) < *δ*, then *λ*_*i*_=(*δ* − (*μ*Λ/(1+*ν*Λ)))^(1/*mα*_2_)^*cis*(*π*/*mα*_2_) for *i*=1,2,…, *mα*_2_ is obtained from *λ*^*mα*_2_^=(*δ* − (*μ*Λ/(1+*ν*Λ)))*cisπ* by De Moivre formulas. In this respect, the stability condition is *α*_2_ < 2 by |arg(*λ*)|=|(*π*/*mα*_2_)| > (*π*/2*m*). This condition has been always provided from (16). Additionally, If (*μ*Λ/(1+*ν*Λ)) ≥ *δ*, then the eigenvalues are positive real number due to *λ*_*i*_=((*μ*Λ/(1+*ν*Λ)) − *δ*)^(1/*mα*_2_)^*cis*0 for *i*=1,2,…, *mα*_2_. In this sense, we have arg(*λ*_*i*_)=0. The stability condition is not provided due to |arg(*λ*)|=0 < (*π*/2*m*). Therefore, the equilibrium point *E*_1_(Λ, 0) is an unstable point for system (16).

Consequently, if the inequality

(33) $$\begin{array}{c} \frac{\mu \Lambda }{1+\nu \Lambda }<\delta \end{array}$$

is provided, then the equilibrium point *E*_1_(Λ, 0) is LAS, and if ((*μ*Λ/(1+*ν*Λ)) − *δ*) ≥ 0, then this point is an unstable point for system (16). When (33) is rearranged, the stability condition of *E*_1_(Λ, 0) is

(34) $$\begin{array}{c} \frac{\delta }{\mu -\nu \delta }>\Lambda . \end{array}$$

(c) Jacobian matrix evaluated at *E*_2_(*P*^*∗*^=*δ*/(*μ* − *νδ*), *T*^*∗*^=*β*_*P*_(1 − (*P*^*∗*^/Λ))/*c*/(1+*aP*^*∗*^)) is
  (35) $$\begin{array}{c} J\left(E_{2}\right)=\left(\begin{array}{cc} \beta_{\text{P}}\left(1-\frac{2P^{*}}{\Lambda }\right)+\frac{T^{*}c}{aP^{*}+1}\left(\frac{aP^{*}}{aP^{*}+1}-1\right) & -\frac{cP^{*}}{aP^{*}+1}\\ \frac{T^{*}\mu }{{\left(1+\nu P^{*}\right)}^{2}} & 0 \end{array}\right), \end{array}$$
  that is,
  (36) $$\begin{array}{c} J\left(E_{2}\right)=\left(\begin{array}{cc} \beta_{P}\left(A_{1}\left(A_{2}+1\right)-1\right) & -\frac{c}{a}A_{2}\\ \frac{a\beta_{P}\delta^{2}A_{1}}{c\mu \Lambda \left(1-A_{1}\right)A_{2}} & 0 \end{array}\right), \end{array}$$
  where *A*_1_ and *A*_2_ are defined in (24).

Let us give more details for *A*_1_ and *A*_2_. Accordingly, it is

(37) $$\begin{array}{c} 0<A_{1}<1, \end{array}$$

since the value *P*^*∗*^, the pathogen size may take, is less than or equal to its carrying capacity Λ. In addition, it is

(38) $$\begin{array}{c} 0<A_{2}<1, \end{array}$$

due to (17), and the components of the equilibrium point in *E*_2_ are positive.

From (36), we have the characteristical equation as follows:

(39) $$\begin{array}{c} \lambda^{m\left(\alpha_{1}+\alpha_{2}\right)}-\lambda^{m\alpha_{2}}\beta_{P}\left(A_{1}\left(A_{2}+1\right)-1\right)+\frac{\beta_{P}\delta^{2}A_{1}}{\mu \Lambda \left(1-A_{1}\right)}=0. \end{array}$$

To be LAS of *E*_2_, it should be that all roots *λ*_*i*_ for *i*=1,2,…, *m*(*α*_1_+*α*_2_) found from the (39) satisfy the inequalities |arg(*λ*_*i*_)| > (*π*/2*m*) in (9).

Proposition is proved.

**Corollary 1 .**

Equation (39) can be examined in more detail as shown below. This equation can be rewritten by De Moivre formulas such that

(40) $$\begin{array}{c} \lambda =rcis\theta =\cos\theta +i\sin\theta ,\\ \lambda^{m\alpha_{1}}=r^{m\alpha_{1}}cism\alpha_{1}\theta ,\\ \lambda^{m\alpha_{2}}=r^{m\alpha_{2}}cism\alpha_{2}\theta , \end{array}$$

where *r* ∈ *ℝ*^+^, angle *θ* ∈ [0,2*π*), and $i=\sqrt{-1}$. By (40), (39) transforms to

(41) $$\begin{array}{c} \left(r^{m\left(\alpha_{1}+\alpha_{2}\right)}cism\left(\alpha_{1}+\alpha_{2}\right)\theta -\beta_{P}\left(A_{1}\left(A_{2}+1\right)-1\right)r^{m\alpha_{2}}cism\alpha_{2}\theta \right)+\frac{\beta_{P}\delta^{2}A_{1}}{\mu \Lambda \left(1-A_{1}\right)}=0, \end{array}$$

and so,

(42) $$\begin{array}{c} \left(r^{m\left(\alpha_{1}+\alpha_{2}\right)}\cos m\left(\alpha_{1}+\alpha_{2}\right)\theta -\beta_{P}\left(A_{1}\left(A_{2}+1\right)-1\right)r^{m\alpha_{2}}\cos m\alpha_{2}\theta +\frac{\beta_{P}\delta^{2}A_{1}}{\mu \Lambda \left(1-A_{1}\right)}\right)+i\left(r^{m\left(\alpha_{1}+\alpha_{2}\right)}\sin m\left(\alpha_{1}+\alpha_{2}\right)\theta -\beta_{P}\left(A_{1}\left(A_{2}+1\right)-1\right)r^{m\alpha_{2}}\sin m\alpha_{2}\theta \right)=0. \end{array}$$

By arranging (42), there is the following system:

(43) $$\begin{array}{c} r^{m\left(\alpha_{1}+\alpha_{2}\right)}\sin m\left(\alpha_{1}+\alpha_{2}\right)\theta -\beta_{P}\left(A_{1}\left(A_{2}+1\right)-1\right)r^{m\alpha_{2}}\sin m\alpha_{2}\theta =0,\\ r^{m\left(\alpha_{1}+\alpha_{2}\right)}\cos m\left(\alpha_{1}+\alpha_{2}\right)\theta -\beta_{P}\left(A_{1}\left(A_{2}+1\right)-1\right)r^{m\alpha_{2}}\cos m\alpha_{2}\theta +\frac{\beta_{P}\delta^{2}A_{1}}{\mu \Lambda \left(1-A_{1}\right)}=0, \end{array}$$

and so,

(44) $$\begin{array}{c} \sin m\left(\alpha_{1}+\alpha_{2}\right)\theta -\beta_{P}\left(A_{1}\left(A_{2}+1\right)-1\right)r^{-m\alpha_{1}}\sin m\alpha_{2}\theta =0,\\ \cos m\left(\alpha_{1}+\alpha_{2}\right)\theta -\beta_{P}\left(A_{1}\left(A_{2}+1\right)-1\right)r^{-m\alpha_{1}}\cos m\alpha_{2}\theta +\frac{\beta_{P}\delta^{2}A_{1}}{\mu \Lambda \left(1-A_{1}\right)}r^{-m\left(\alpha_{1}+\alpha_{2}\right)}=0. \end{array}$$

From the first equation in system (44), we have found

(45) $$\begin{array}{c} r={\left(\frac{\beta_{P}\left(A_{1}\left(A_{2}+1\right)-1\right)\sin m\alpha_{2}\theta }{\sin m\left(\alpha_{1}+\alpha_{2}\right)\theta }\right)}^{1/m\alpha_{1}}. \end{array}$$

By substituting (45) in the second equation in (44), it is found

(46) $$\begin{array}{c} \frac{{\left(\sin m\alpha_{2}\theta \right)}^{\left(\alpha_{2}/\alpha_{1}\right)}\sin m\alpha_{1}\theta }{{\left(\sin m\left(\alpha_{1}+\alpha_{2}\right)\theta \right)}^{\left(1+\left(\alpha_{2}/\alpha_{1}\right)\right)}}=\frac{\left(\beta_{P}\delta^{2}A_{1}\right)/\left(\mu \Lambda \left(1-A_{1}\right)\right)}{{\left(\beta_{P}\left(A_{1}\left(A_{2}+1\right)-1\right)\right)}^{\left(1+\left(\alpha_{2}/\alpha_{1}\right)\right)}}. \end{array}$$

Consequently, if the angles *θ*_*i*_ for *i*=1,2,…, *m*(*α*_1_+*α*_2_) obtained from (46) satisfy Routh–Hurwitz stability criteria ((*π*/2) < *θ* < *π*) or the condition (9) (|*θ*| > (*π*/2*m*)), then *E*_2_ is LAS.

For equilibria of system (16), the conditions found for LAS and biological existence are summarized in Table 2.

**Corollary 2 .**

*E*_1_ is an unstable point, when *E*_2_ exists biologically. Therefore, these equilibrium points cannot be stable when together. Similarly, *E*_2_ is biologically meaningless, when *E*_1_ is LAS. These circumstances appeared are also seen in Table 2.

**Corollary 3 .**

Let us consider the special case of *α*_1_=*α*_2_=*α* for *E*_2_. In this case, we have Remark 4. The characteristical equation obtained from Det(*J*(*E*_2_) − *λI*_2_)=0 is

(47) $$\begin{array}{c} \lambda^{2}-\lambda \beta_{P}\left(A_{1}\left(A_{2}+1\right)-1\right)+\frac{\beta_{P}\delta^{2}A_{1}}{\mu \Lambda \left(1-A_{1}\right)}=0. \end{array}$$

The conditions for LAS of the equilibrium point *E*_2_ are either Routh–Hurwitz stability conditions.

(48) $$\begin{array}{c} A_{1}\left(A_{2}+1\right)-1<0, \end{array}$$

due to (37) and (38), or the conditions

(49) $$\begin{array}{c} \left\{\begin{array}{c} \left(A_{1}\left(A_{2}+1\right)-1\right)>0,\\ 4\frac{\beta_{P}\delta^{2}A_{1}}{\mu \Lambda \left(1-A_{1}\right)}>{\left(A_{1}\left(A_{2}+1\right)-1\right)}^{2},\\ \left|\tan^{-1}\left(\frac{\sqrt{4\left(\left(\beta_{P}\delta^{2}A_{1}\right)/\left(\mu \Lambda \left(1-A_{1}\right)\right)\right)-{\left(A_{1}\left(A_{2}+1\right)-1\right)}^{2}}}{\left(-\left(A_{1}\left(A_{2}+1\right)-1\right)\right)}\right)\right|>\frac{\alpha \pi }{2}, \end{array}\right\}. \end{array}$$

In addition that, by considering equation (46), there may be found a similar condition for stability of this point. In this sense, it is

(50) $$\begin{array}{c} \theta =\frac{1}{2m\alpha }\cos^{-1}\left(\frac{\beta_{P}{\left(\left(A_{1}\left(A_{2}+1\right)-1\right)\right)}^{2}}{2\left(\left(\delta^{2}A_{1}\right)/\left(\mu \Lambda \left(1-A_{1}\right)\right)\right)}-1\right), \end{array}$$

by formulas of half angle. Thereby, the LAS condition of *E*_2_ is

(51) $$\begin{array}{c} \left|\cos^{-1}\left(\frac{\beta_{P}{\left(\left(A_{1}\left(A_{2}+1\right)-1\right)\right)}^{2}}{2\left(\left(\delta^{2}A_{1}\right)/\left(\mu \Lambda \left(1-A_{1}\right)\right)\right)}-1\right)\right|>\pi \alpha , \end{array}$$

where −1 ≤ (*β*_*P*_((*A*_1_(*A*_2_+1) − 1))^2^/2((*δ*^2^*A*_1_)/(*μ*Λ(1 − *A*_1_)))) − 1 ≤ 1. Corollary 3 is summarized in Table 3.

## 4. Numerical Analysis for Model

The proposed model in this study summarizes the general dynamics of the pathogen-immune system. The immune system cells described herein are memory T cells specially produced by the host against the pathogen. Hence, the proposed model is suitable for modeling of diseases such as chronic infections or tumors in which the nonspecific immune system cells of the host at the beginning of the disease have failed to destroy the pathogen.

The conditions found in Table 2 have been supported by numerical studies shown below. In this section, the time-dependent sizes of the tumor and memory T cells for cancer tried to be estimated by giving the different values to the parameters in the proposed model. The reason for this is to be able to obtain different scenarios and to better demonstrate the results of qualitative analysis. The values of parameters used in system (16) are shown in Table 4.

The values calculated by Table 4 are given in Table 5.

**Figure 2:**
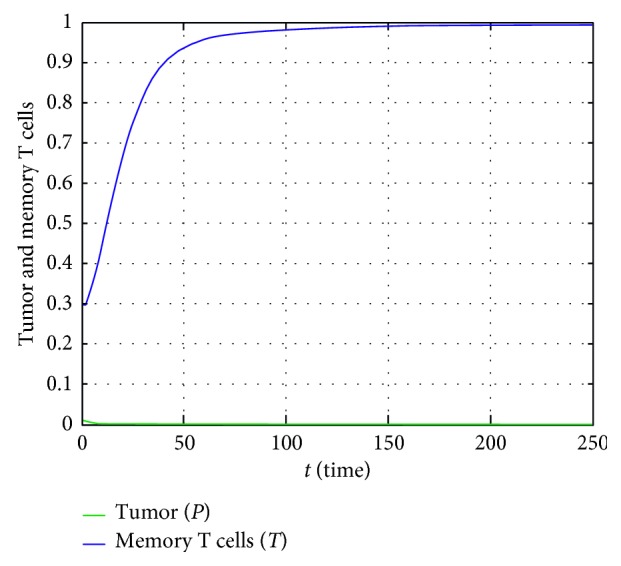
In case of the first column data in Table 4, temporary course of population size of the tumor and memory T cells.

Through the values in the first columns of Table 4, the stability of the equilibrium point *E*_1_(1,0), where the tumor exists and it approaches its carrying capacity, is obtained as shown in Figure 2. This happens within at least 200 days.

**Figure 3:**
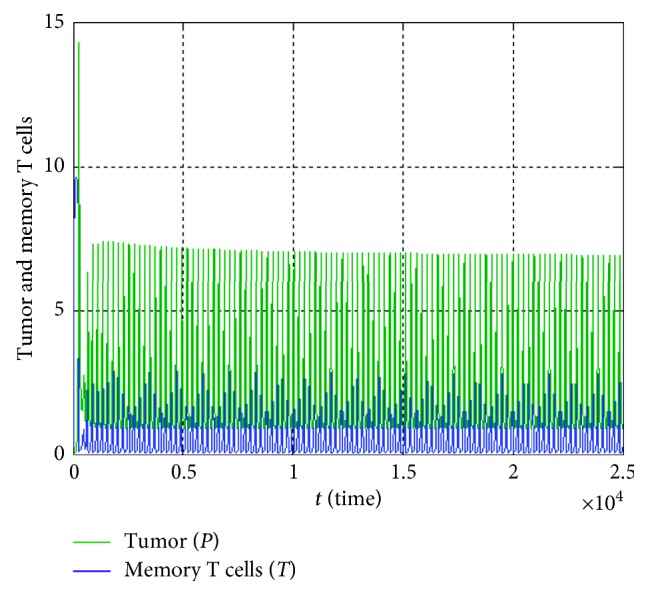
In case of the second column data in Table 4, temporary course of population size of the tumor and memory T cells.

When the values in the second columns of Table 4 are used, a situation where the system behaviour is limit cycle and *E*_2_(*P*^*∗*^, *T*^*∗*^) is an unstable point is obtained as shown in Figure 3.

**Figure 4:**
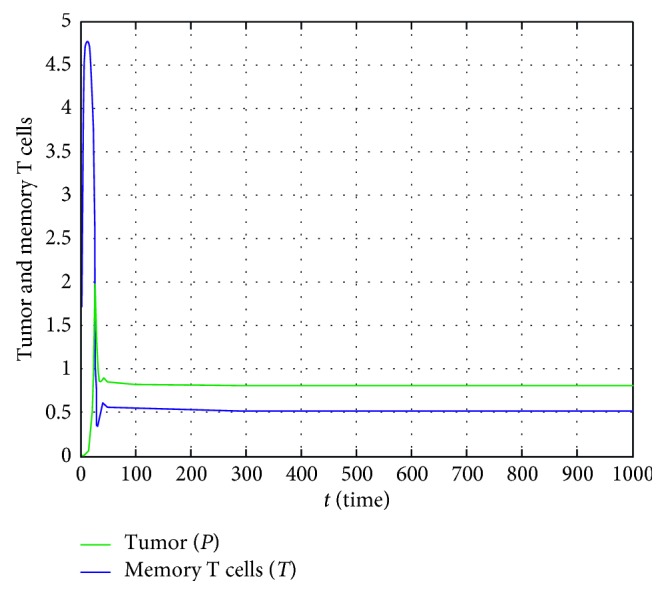
In case of the third column data in Table 4, temporary course of population sizes of the tumor and memory T cells.

Finally, let us consider the third column in Table 4. In here, *P*^*∗*^ for the tumor has a value of 0.5 and *T*^*∗*^ for memory T cells has a value of 0.81. This occurs in at least 100 days as seen in Figure 4. Therefore, these two types of cells stay permanently in the host.

## 5. Conclusions

In this study, the mathematical model examining the changes in the pathogen population size under pressure of specific immune system response in case of cancer or chronic infection has been constructed by the FODE system with multi-orders. According to the results of analysis of model, the pathogen causing disease never disappears in host unless an additional treatment is provided, since the disease is continued by a pathogen, and the free-disease equilibrium point *E*_0_(0,0) is an unstable point. This case is very suitable for the presumed diseases in the proposed model.

When the existence condition of *E*_2_(*P*^*∗*^, *T*^*∗*^) and the stability condition of *E*_1_(Λ, 0) in Table 2 is rearranged, then

(52) $$\begin{array}{c} \delta <\frac{\mu \Lambda }{1+\nu \Lambda },\\ \delta >\frac{\mu \Lambda }{1+\nu \Lambda }, \end{array}$$

is obtained, respectively. The term (*μ*Λ/(1+*ν*Λ)) is the rate of growth of the memory T cells when the tumor reaches its carrying capacity Λ. The parameter *δ* is the natural death rate of memory T cells. Accordingly, the value (((*μ*Λ)/(1+*ν*Λ)) − *δ*) can be interpreted as the proliferative power of the memory T cells against the tumor. When this value is negative as seen in Figure 2, the memory T cells cannot reproduced as enough, and consequently, it can be mentioned from the stability of the equilibrium point *E*_1_, that only the tumor exists, and the tumor approaches its carrying capacity. Let us consider that this value is positive, and some additional conditions in Table 2 are met. As can be seen in Figure 4, it can be said that the positive equilibrium point *E*_2_, in which both tumor and memory T cells exist, is stable.

In numerical studies, we tried to estimate the timing and magnitude of the development of the tumor. If treatment procedure for the individual has not been applied, then the results obtained from the proposed model emphasize the fact that either the tumor reaches its maximum size and the memory T cells collapse or the tumor and memory T cells continue to stay together in the host. In the last case mentioned above that the memory T cells of the individual does not collapse, the tumor maintains its presence in the host in a limited manner. The results obtained from analysis are quite consistent with the scenarios of real situations related to the tumor.

## Figures and Tables

**Table 1:** Biological existence conditions for the equilibria of system (16).

Equilibrium points	Biological existence conditions
*E*_0_(0,0)	Always exists
*E*_1_(Λ, 0)	
*E*_2_(*P*^*∗*^, *T*^*∗*^)	*δ*/(*μ* − *νδ*) < Λ

**Table 2:** LAS and biological existence conditions for the equilibria of system (16).

Equilibrium points	Biological existence conditions	LAS conditions
*E*_0_(0,0)	Always exists	Unstable point
*E*_1_(Λ, 0)	Always exists	(*δ*/(*μ* − *νδ*)) > Λ
*E*_2_(*P*^*∗*^, *T*^*∗*^)	(*δ*/(*μ* − *νδ*)) > Λ	For *i*=1,2,…, *m*(*α*_1_+*α*_2_), either the eigenvalues *λ*_*i*_ obtained from the characteristic equation *λ*^*m*(*α*_1_+*α*_2_)^ − *λ*^*mα*_2_^*β*_*P*_(*A*_1_(*A*_2_+1) − 1)+((*β*_*P*_*δ*^2^*A*_1_)/(*μ*Λ(1 − *A*_1_)))=0 satisfy |arg(*λ*)| > (1/*m*)(*π*/2) or the angles *θ*_*i*_ obtained from the equation (((sin*mα*_2_*θ*)^(*α*_2_/*α*_1_)^sin*mα*_1_*θ*)/((sin*m*(*α*_1_+*α*_2_)*θ*)^(1+(*α*_2_/*α*_1_))^))=(*β*_*P*_*δ*^2^*A*_1_/*μ*Λ(1 − *A*_1_))/(*β*_*P*_(*A*_1_(*A*_2_+1) − 1))^(1+(*α*_2_/*α*_1_))^ satisfy |*θ*_*i*_| > (*π*/2*m*)

where (*P*^*∗*^, *T*^*∗*^) and (*A*_1_, *A*_2_) are defined in (19) and (24), respectively.

**Table 3:** The LAS conditions for *E*_2_(*P*^*∗*^, *T*^*∗*^), in case of *α*_1_=*α*_2_=*α*.

LAS conditions of *E*_2_
*A*_1_(*A*_2_+1) − 1 < 0 (from Routh–Hurwitz criteria in (14))
Or
$\left\{\begin{array}{c} \left(A_{1}\left(A_{2}+1\right)-1\right)>0,\\ 4\left(\left(\beta_{P}\delta^{2}A_{1}\right)/\left(\mu \Lambda \left(1-A_{1}\right)\right)\right)>{\left(A_{1}\left(A_{2}+1\right)-1\right)}^{2},\\ \left|\tan^{-1}\left(4\left(\beta_{P}\delta^{2}A_{1}\right)/\left(\mu \Lambda \left(1-A_{1}\right)\right)-{\left(A_{1}\left(A_{2}+1\right)-1\right)}^{2}\right)/\left(-\left(A_{1}\left(A_{2}+1\right)-1\right)\right)\right|>\left(\alpha \pi /2\right), \end{array}\right\}$ (from |arg(*λ*_*i*_)| > (*απ*/2) in (15))
Or
|cos^−1^((*β*_*P*_((*A*_1_(*A*_2_+1) − 1))^2^/2(*δ*^2^*A*_1_/*μ*Λ(1 − *A*_1_))) − 1)| > *πα* (from (46))

**Table 4:** The interpretation and considered values of the parameters in the proposed model.

Parameters	Descriptions	Units	Values		
			For Figure 2	For Figure 3	For Figure 4
*β*_*P*_	Growth rate of the tumor	Day^−1^	2.4	2.4	2.4
Λ	Carrying capacity of the tumor	Cells	1	10	5
*c*	Maximum killing rate of the tumor by immune cells	Day^−1^	4	4	4
*a*	Immune cells for half maximum effect on the tumor	Cell^−1^·day^−1^	0.2	4	1
*μ*	The effect of capture rate of immune cells	Day^−1^	3.98	3.9	3.9
*ν*	The tumor population size at which the growth rate of immune cells is half its maximum	Cell^−1^·day^−1^	1.9	1.9	1.9
*δ*	Natural death rate of immune cells	Day^−1^	1.99	1	1
*α*_1_	Fractional-order of the first equation in (16)	A rational number in the interval (0,1|	0.9	0.8	0.8
*α*_2_	Fractional-order of the second equation in (16)	A rational number in the interval (0,1|	0.75	0.6	0.6

**Table 5:** The values calculated from Table 4 according to Table 2.

Expressions	Terms	Values		
		For Figure 2	For Figure 3	For Figure 4
Equilibrium point *E*_1_	*E*_1_(Λ, 0)	*E*_1_(1,0)	*E*_1_(10,0)	*E*_1_(5,0)
Stability condition of *E*_1_	(*δ*/*μ* − *νδ*) > Λ	10 > 1 (*E*_1_ is LAS)	0.50 < 10 (*E*_1_ is unstable)	0.50 < 5 (*E*_1_ is unstable)
Equilibrium point *E*_2_	*E*_2_(*P*^*∗*^=*δ*/(*μ* − *νδ*), *T*^*∗*^=*β*_*P*_(1 − (*P*^*∗*^/Λ))/(*c*/(1+*aP*^*∗*^)))	*E*_2_(10, −16.20)(biologically meaningless)	*E*_2_(0.50, 1.71)	*E*_2_(0.50, 0.81)
Parameter *A*_1_	(1 − (*P*^*∗*^/Λ))	—	0.95	0.90
Parameter *A*_2_	(*aP*^*∗*^/*aP*^*∗*^+1)	—	0.66667	0.3333
Least common multiple of order's denominator	*m*	—	5	5
Characteristical equation of eigenvalues for *E*_2_	*λ* ^*m*(*α*_1_+*α*_2_)^ − *λ*^*mα*_2_^*β*_*P*_(*A*_1_(*A*_2_+1) − 1)+((*β*_*P*_*δ*^2^*A*_1_)/(*μ*Λ(1 − *A*_1_)))=0	—	*λ* ^7^ − 1.40*λ*^3^+1.1692=0	*λ* ^7^ − 0.48*λ*^3^+1.1077=0
The eigenvalues for *E*_2_	—	—	*λ*_1_ ≈ 0.9614+0.2454*i* *λ*_2_ ≈ 0.9614 − 0.2454*i* *λ*_3_ ≈ 0.1199+1.1495*i* *λ*_4_ ≈ 0.1199 − 1.1495*i* *λ*_5_ ≈ −1.2004 *λ*_6_ ≈ −0.4812+0.7135*i* *λ*_7_ ≈ −0.4812 − 0.7135*i*	*λ*_1_ ≈ 0.9287+0.3757*i* *λ*_2_ ≈ 0.9287 − 0.3757*i* *λ*_3_ ≈ 0.1852+1.0410*i* *λ*_4_ ≈ 0.1852 − 1.0410*i* *λ*_5_ ≈ −1.0798 *λ*_6_ ≈ −0.5741+0.7646*i* *λ*_7_ ≈ −0.5741 − 0.7646*i*
Angle of eigenvalues for *E*_2_	*θ*=arg(*λ*)	—	*θ*_1_ ≈ 14.3191°, *θ*_2_ ≈ −14.3191°, *θ*_3_ ≈ 84.0452°, *θ*_4_ ≈ −84.0452°, *θ*_5_ ≈ 180°, *θ*_6_ ≈ 123.997°, *θ*_7_ ≈ −123.997°	*θ*_1_ ≈ 22.0255°, *θ*_2_ ≈ −22.0255°, *θ*_3_ ≈ 79.9123°, *θ*_4_ ≈ −79.9123°, *θ*_5_ ≈ 180°, *θ*_6_ ≈ 126.901°, *θ*_7_ ≈ −126.901°
Stability condition of *E*_2_	|*θ*_*i*_| > (*π*/2*m*)	—	*E*_2_ is an unstable point, since |*θ*_1_|, |*θ*_2_| ≈ 14.31° < 18°.	*E*_2_ is LAS, since |*θ*_1_|, |*θ*_2_|,…, |*θ*_7_| > 18°.
Initial conditions	(*P*_0_, *T*_0_)	(0.3, 0.01)	(0.3, 0.01)	(0.3, 0.01)

## Data Availability

The data used to support the findings of this study are available from the corresponding author upon request.
